# Snake Poisons and Antidotes

**Published:** 1895-07-06

**Authors:** 


The Hospital

An Institutional Journal of

The Medical Sciences and Hospital Administration, with

Vol. XVIII.—No. 458.] AN EXTRA NURSING SUPPLEMENT. [July 6, 1895.

### Snake Poisons and Antidotes.

Professor Frazer's experiments with snake poisons have a dual importance at the present time. They are important in that if successful they will establish a method which may save many thousands of lives in India, where now more than 20,000 persons are annually killed by snake bites. But they are almost equally, or possibly even more, important as confirming the generalisation which is now being formed, and which is being based on a series of proved results, showing the possibility of immunisation against manifold series of deadly poisons to which human beings and animals all the world over are exposed.

The experiments are being carried out on an extensive scale; as, indeed, if they are to command confidence they must be. Hundreds of experimenters have done their best to bring physiological research into contempt by the limited range of their work and the pettifogging nature of their methods. A dozen mice, a few solutions, a little more or less careful dissection have constituted the stock-in-trade of these experimental philosophers; and what they have lacked in knowledge, material resources, and philosophical capacity for research they have made up for by unlimited assurance and a persistence of assertion which have wearied and disgusted the scientific world. But men's eyes are gradually being opened; not only the eyes of the medical profession, but of journalists and general writers and thinkers of every class. Those who aspire to enter upon the path of original research in the future must make up their minds to run the gauntlet of a widely-informed and competent criticism such as ten years ago was not so much as dreamed of.

Professor Frazer has an adequate comprehension of the rational and indispensable conditions of genuine original research. He has, either personally or by his agents, placed himself in communication with India, America, Australia, and Africa. That is to say, he has, as far as possible, dealt with actual snake poisons, as produced under the ordinary conditions of natural snake life. It is obvious that one of the least satisfactory of all methods of investigation would have been to have commenced operations with excited snake-bitten persons at the moment of their terror and danger. At such a time all is hurry and excitement. Calm observation, leisurely weighing of facts, unprejudiced reasoning are impossible. The laboratory is the place where alone investigations can be adequately commenced and philosophical conclusions reached. It is in the confirmation, or otherwise, of such conclusions, that experiments on the actual field of battle—that is, with actual snakes and snake-bitten persons—are so invaluable and indispensable.

The experiments of Professor Frazer have as yet been confined to the laboratory. But they have been made with actual snake poisons, and with these poisons in the condition of demonstrated deadly activity. Incidentally, many interesting facts have been brought to light, though the ultimate goal of the experimentations has never been lost sight of. It has been shown, for example, that not only is there a difference of deadliness in the poison of certain snakes, but that the poison of one and the same snake may be much less deadly at certain times and under certain conditions than at other times and under other conditions. All this, which may appear to be but incidental to the main lines of research, will be found to be of first-rate importance when immunisation and curative treatment come to be applied in practice.

The objects of Professor Frazer in his researches have been twofold: first, he has aimed at producing artificial immunity, and, secondly, at discovering and separating a substance which will cure persons actually inoculated with snake poison by snake bites. To have acquired the power of rendering human beings immune against snake poison would be an interesting step in the onward march of science; and there are many conceivable circumstances under which the possession of such a power might be of very great practical importance. But it is an antidote against snake poisons that the medical profession and the general public are most eagerly looking for in this connection, and it is as a possible antidote that the new discovery will make its most powerful appeal to ordinary men and women. Has Professor Frazer succeeded in providing us with a new cure for snake bites? And may the cure be trusted if any of us should unfortunately require to put it to the proof? These are the questions the answers to which must decide the Professor's claim to have made an important and lasting contribution to medical and general science.

It seems to have been experimentally established by Professor Frazer that *antivenene*, as he calls his new antidote, and which consists of the blood serum of immunised animals in the form of a dry and portable powder, has certain definite but strictly limited antidotal powers against snake poisons, introduced into the animal organism by artificial means. For example, in a series of experiments made with lethal doses of cobra and other snake poisons, recovery took place as the result of the injection of antivenene as long as thirty minutes after inoculation with the poison. This, it will be noted, is a result of some value. It seems clear, however, that if treatment cannot be immediate there is little hope of cure; moreover, a very large dose of venom from a large and very angry snake would appear to demand, not only treatment within a few minutes of the bite, but so large a dose of the antivenene as might itself prove dangerous. In summarisation, therefore, all that can be said at the present time is that Professor Frazer has undertaken a research of great importance, and that there is a promise of good results. We are glad to know that Dr. Calmette, head of the bacteriological station at Saïgon, in Cochin China, is an experienced and philosophical worker in the same field of research.

